# Influence of housing damage caused by the Great East Japan Earthquake on the association between new social isolation and depressive symptoms during the COVID-19 pandemic: findings from the Tohoku Medical Megabank Community-Based Cohort Study

**DOI:** 10.1186/s12889-025-25996-9

**Published:** 2025-12-24

**Authors:** Yuka Kotozaki, Kozo Tanno, Kotaro Otsuka, Makoto Sasaki

**Affiliations:** 1 Iwate Tohoku Medical Megabank Organization, Iwate Medical University, Iwate, Japan; 2 Department of Hygiene and Preventive Medicine, School of Medicine, Iwate Medical University, Iwate, Japan; 3 Department of Neuropsychiatry, School of Medicine, Iwate Medical University, Iwate, Japan; 4 Division of Ultrahigh Field MRI, Institute for Biomedical Sciences, Iwate Medical University, Iwate, Japan

**Keywords:** Coronavirus disease 19, Social isolation, Past disaster experience, Depressive symptoms

## Abstract

**Background:**

It is unclear whether past disaster experience, specifically housing damage caused by the Great East Japan Earthquake (GEJE), influenced the association between social isolation and depressive symptoms during the coronavirus disease 2019 (COVID-19) pandemic. Therefore, we aimed to examine the association between new social isolation and depressive symptoms during the COVID-19 pandemic and whether this association was influenced by past disaster experience.

**Methods:**

We analyzed the longitudinal data of 8,647 individuals, excluding those who reported social isolation before COVID-19. The presence of social isolation and depressive symptoms were defined using the Lubben Social Network Scale-6 (LSNS-6) score < 12 and the Center for Epidemiological Studies-Depressive Scale (CES-D) score ≥ 16, respectively. Pre-COVID-19 survey was conducted between June 2017 and February 2020, and COVID-19 survey was conducted between November 2020 and March 2021. Participants were categorized into “not socially isolated” and “newly socially isolated” groups. Logistic regression analyses were performed to calculate the multivariable-adjusted odds ratio (AOR) with 95% confidence interval (CI) for depressive symptoms, comparing both groups.

**Results:**

Participants who were newly socially isolated during the COVID-19 pandemic had a significantly higher prevalence of depressive symptoms than those who were not socially isolated in both men and women (men: AOR 1.53 [CI: 1.15–2.02]; women: AOR 1.96 [CI: 1.64–2.34]). However, past disaster experience did not affect this association in both men and women.

**Conclusions:**

To reduce depressive symptoms during a major infectious disease pandemic such as the COVID-19 pandemic, it is important to adopt a public health perspective to prevent social isolation.

**Supplementary Information:**

The online version contains supplementary material available at 10.1186/s12889-025-25996-9.

## Background

Coronavirus disease 2019 (COVID-19), first reported in late December 2019 in Wuhan, Hubei Province, China, has quickly spread worldwide. On January 30, 2020, the World Health Organization (WHO) declared it a “public health emergency of international concern” [1], and for three years and four months until May 5, 2023, when the WHO announced the end of the declaration, countries worldwide were forced to strengthen their infection control measures [2]. In many countries, social distancing and refraining from going out unless urgent were promoted to control the spread of COVID-19 [3]. The sudden and prolonged restrictions on behavior weakened social ties and raised concerns about social isolation [4]. Social isolation refers to a lack of social contact or support [5]. It is generally acknowledged as a major public health problem and is associated with detrimental health outcomes [6, 7], including increased risk of poor mental health, cognitive decline, and mortality [8, 9].

**Fig. 1:**
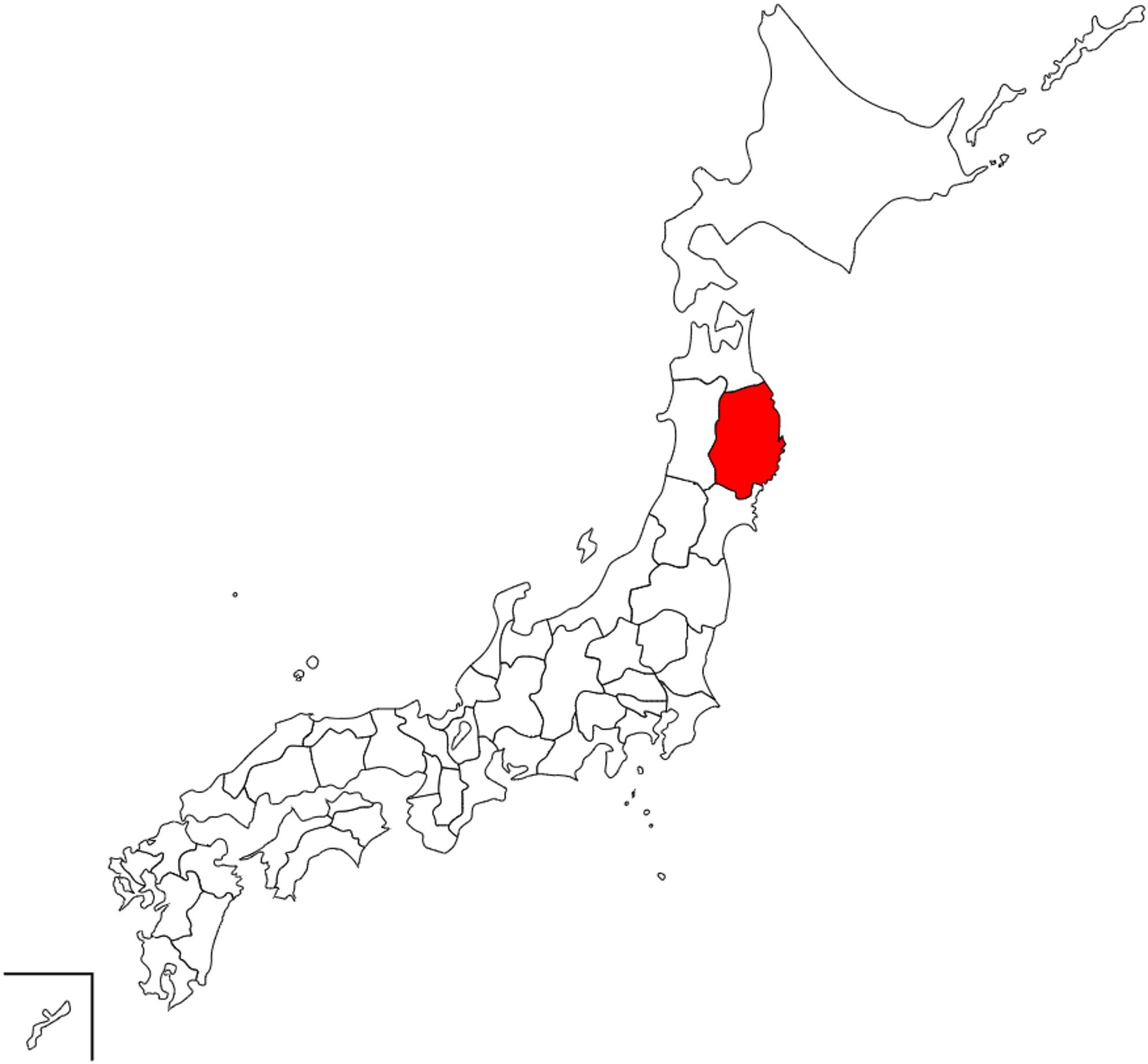
Geographic location of Iwate Prefecture, Japan

Around the same time as the COVID-19 spread in China, several cases were reported in Japan, with Iwate Prefecture being one of the areas with the slowest outbreaks in Japan [10]. Iwate Prefecture is located in Japan’s Tohoku region and along the Pacific coast (Fig. 1). This is because it has one of the lowest population densities in Japan, resulting in relatively little human contact and, invariably, a low risk of COVID-19 [10]. In addition, the relatively low traffic volume and small number of people traveling in the city compared with other prefectures and large cities made the spread of COVID-19 less likely [10]. During our data collection period (November 2020 to February 2021), COVID-19 prevention measures in Iwate Prefecture, including social distancing, restrictions on large gatherings, and temporary closures of schools and public facilities, were in place. These measures may have affected social interactions and should be considered when interpreting the association between social isolation and depressive symptoms.

Studies conducted early during the COVID-19 pandemic showed increased prevalence rates of social isolation [4, 11, 12] and depressive symptoms [11, 13, 14]. Robb et al. reported that social isolation is associated with depressive symptoms, using data from the Cognitive Health in Aging Register for Interventional and Observation Trials COVID-19 Rapid Response Study conducted among healthy older adults living in London [15]. Pancani et al. also reported that social isolation worsens depressive symptoms, using data from an online survey conducted in Italy [16]. An online survey of 4,823 people in Germany showed an increase in social isolation during compared with before the COVID-19 pandemic [17]. Li et al. studied 2,749 community-dwelling older adults aged 60 years and older before and during the COVID-19 pandemic and suggested that newly and continuously socially isolated groups were more likely to experience worse depressive symptoms after experiencing the COVID-19 pandemic [18].

Past disasters can have enduring effects on individuals’ psychological well-being and social networks, potentially amplifying the risks associated with social isolation during subsequent crises. In Iwate Prefecture, the Great East Japan Earthquake (GEJE) occurred on March 11, 2011, primarily affecting the Tohoku region of Japan and causing widespread destruction and loss of life [19]. The disaster also had long-term social and health impacts, and social isolation was found to be highly associated with depressive symptoms, even before the COVID-19 pandemic [20, 21]. Exposure to the GEJE may influence the relationship between social isolation and depressive symptoms during the COVID-19 pandemic, as previous studies have shown that disaster experiences can have long-term social and psychological impacts, including increased social isolation and heightened vulnerability to depression [20, 21]. Therefore, individuals who experienced housing damage or other severe impacts from the GEJE might be more susceptible to the adverse mental health effects of social isolation during the pandemic.

To investigate the long-term health impacts of the GEJE, the Tohoku Medical Megabank Community-based Cohort Study (TMM CommCohort study) was established in the affected areas of Iwate and Miyagi Prefectures. This cohort was designed to collect comprehensive health information and biospecimens from residents, with the objective of supporting disease prevention and medical research [22, 23]. However, it remains unclear whether past disaster experience influenced the association between social isolation and depressive symptoms during the COVID-19 pandemic.

The objective of this study was threefold. First, we aimed to examine the association between new social isolation during the COVID-19 pandemic and the prevalence of depressive symptoms. Second, we sought to investigate whether housing damage caused by the GEJE moderates this association. Finally, as a sensitivity analysis to check the robustness of the main findings, we examined whether the association between social isolation and depressive symptoms differed according to baseline depressive symptoms.

## Methods

### Study population

The TMM CommCohort Study has been conducted since 2013. For this study, only participants residing in Iwate Prefecture were included. Participants aged 20 years or older, registered in the basic resident register of municipalities in Iwate Prefecture, were recruited primarily through municipal health checkups and direct invitations. Baseline data were collected between 2013 and 2016 [23].

A total of 32,320 of the 32,919 participants from the baseline survey were followed up, excluding six dual enrollees and 597 who withdrew consent. Pre-COVID-19 survey was conducted between June 2017 and February 2020 among 20,809 participants. COVID-19 survey was conducted between November 2020 and February 2021 among 16,688 participants (response rate: 80.2%). Of these, 16,647 had data for both surveys. We excluded 1,055 participants with missing data on the Lubben Social Network Scale (LSNS-6), 2,023 participants with missing data on the Center for Epidemiological Studies-Depression Scale (CES-D), 718 participants with missing data on disaster status (home damage or death in the family due to the GEJE), 599 participants with missing data on other covariates, and 3,605 participants who were socially isolated before the COVID-19 pandemic.

**Fig. 2:**
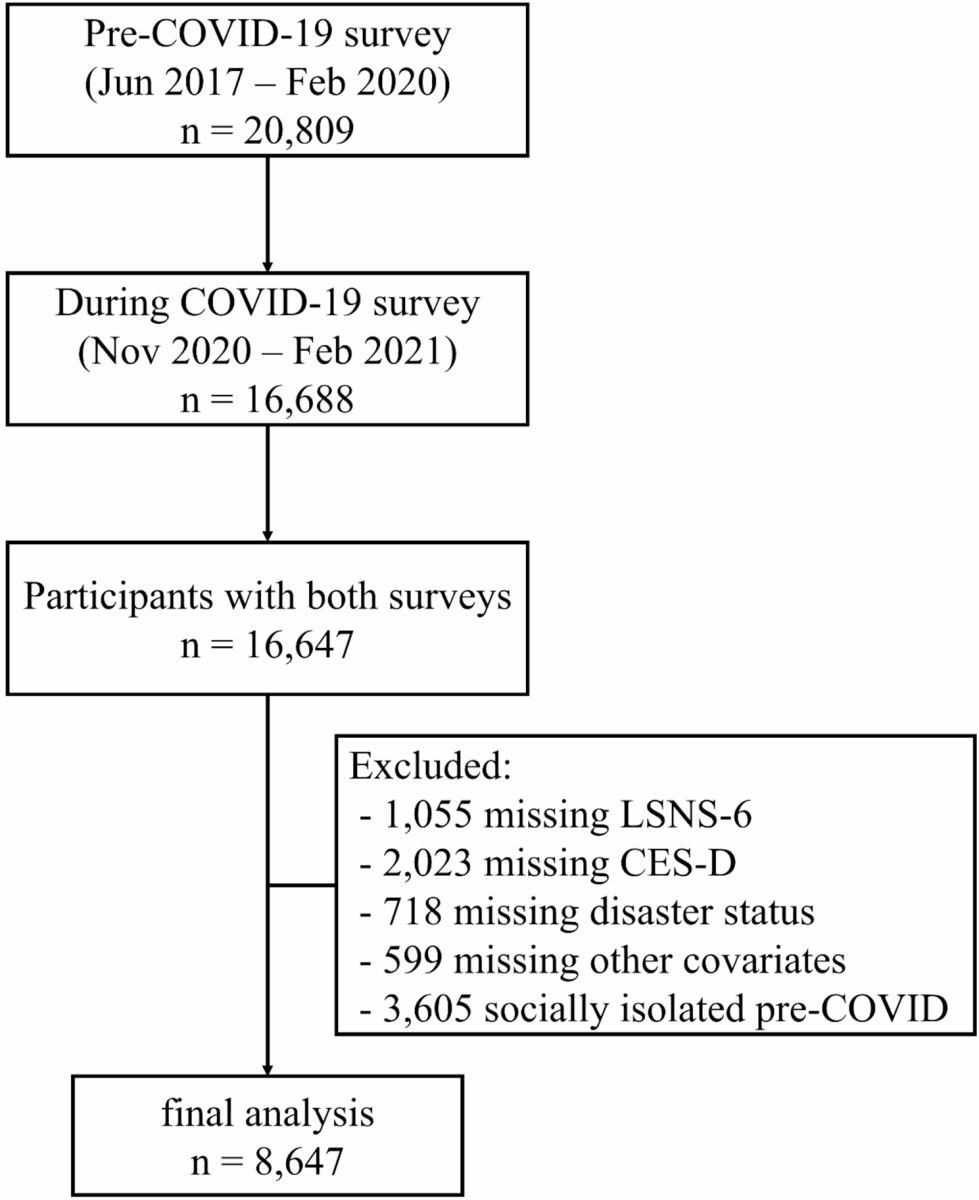
Participant selection flowchart.

Consequently, our final analysis included 8,647 participants (2,881 men and 5,766 women, age 64.4 ± 10.6 years) (Fig. 2). This study was approved by the Ethics Committee of Iwate Medical University (HG H25-2 and HG 2018-004), and all participants provided written informed consent.

### Social isolation before and during the COVID-19 pandemic

Social isolation was assessed using the Japanese version of the LSNS-6 [24], which consists of six items on social connections (three questions about family ties and three about friendship ties). Each item is scored from 0 to 5, yielding a total score ranging from 0 to 30, with a score < 12 indicating social isolation. The LSNS-6 was measured both before and during the COVID-19 pandemic. The Japanese version has demonstrated good reliability, with Cronbach’s α = 0.82.

### Depressive symptoms before and during the COVID-19 pandemic

Depressive symptoms were assessed using the Japanese version of the CES-D [25]. Each of the 20 items is scored 0–3, for a total range of 0–60. A CES-D score ≥ 16 was considered indicative of depressive symptoms. Assessments were conducted before and during the pandemic. The Japanese version of CES-D has demonstrated high reliability, with Cronbach’s α = 0.88.

### House damage caused by the GEJE

GEJE-related house damage was assessed using the following options: (1) totally damaged (including all outflows), (2) seriously damaged, (3) half-damaged, (4) partially damaged, (5) undamaged, and (6) non-residence. For analysis, these were dichotomized into damaged (totally damaged or seriously damaged, half-damaged, or partially damaged) or undamaged (no damage or non-residence), representing substantial direct impacts of the earthquake on participants’ lives.

### Sociodemographic variables

The analysis included the following demographic characteristics: sex (men or women), age (continuous), BMI (< 18.5, 18.5 to < 25.0, ≥ 25.0), education level (elementary and junior high school, high school, university degree, and others), marital status (unmarried or married), number of household members (living alone or ≥ 2), smoking status (nonsmoker or current smoker), drinking status (nondrinker or current drinker), exercise habits (no or yes), working status (not working or working), lack of social capital, insomnia, and depressive symptoms before the COVID-19 pandemic. Of these, sex, education level, and working status were collected before the COVID-19 pandemic, whereas other variables were collected both before and during the COVID-19 pandemic. Lack of social capital was determined by a score < 9 on social capital [26]. Insomnia was determined by a score ≥ 6 on the Athens Insomnia Scale [27]. Lack of social capital and social isolation capture different aspects of social connectedness. Social network is a structural concept that represents who and how many people an individual is connected to, whereas social capital relates to the quality and value of relationships, focusing on the trust and support generated through these connections [28]. Including both variables allows us to account for the potential influence of community-level social support on depressive symptoms, independent of individual-level social isolation.

### Behavior during the COVID-19 pandemic

We assessed abstention from going out by asking the following questions during the COVID-19 survey (2020–2021): “Did you refrain from going out until April 16 (before the first declaration of a state of emergency in Japan took effect)?” “Did you refrain from going out after April 17 (when the nationwide declaration of a state of emergency took effect)?” and “Did you refrain from going out after July 29 (when an infected person was confirmed in Iwate Prefecture)?” Response options were on a five-point scale: refrained from going out, refrained from going out somewhat, neither, not much, and not at all.

Interpersonal relationships were assessed with items asking whether opportunities to meet face-to-face with non-family members, family time, arguments with family, and use of social networking services/phone calls had changed, each on a five-point scale ranging from increased to decreased. Lifestyle situations were assessed by asking whether shopping frequency or income had changed, also with five response options. Each item was coded so that higher scores reflected greater restriction or increase, and variables were used as categorical indicators in the analyses. These behavioral items were developed specifically for this survey to capture pandemic-related changes; therefore, internal consistency indices such as Cronbach’s alpha were not calculated, and each item was treated as an independent categorical variable in the analyses.

### Statistical analysis

Participants were classified based on their LSNS-6 scores, with a score of < 12 indicating social isolation. Social isolation was measured at two time points: pre-pandemic (2017–2020) and during the COVID-19 pandemic (2020–2021). Participants were categorized into two groups: those who were not socially isolated at either time point (persistent non-isolated) and those who were not isolated pre-pandemic but became isolated during the pandemic (newly socially isolated). Participants who were persistently socially isolated, representing a chronic rather than acute exposure, were excluded from the main analysis because the study focused on the effects of newly developed social isolation during the pandemic. Differences in participant characteristics between the two groups were assessed using the chi-square test.

To examine the influence of behavior during the COVID-19 pandemic on the association between new social isolation and depressive symptoms, a correlation analysis of questionnaire items regarding pandemic-related behavior was conducted (see Supplementary Table 1, Additional File 1). Among the three items concerning refraining from going out, which showed moderately high correlations with one another, “Refraining from going out after July 29, when the first COVID-19 case was confirmed in Iwate Prefecture,” was selected as a representative adjustment factor. This adjustment was performed to account for potential confounding, as behavior changes such as staying at home may influence both social isolation and depressive symptoms.

Sex differences in the association between social isolation and depressive symptoms have been reported in previous studies [20, 29], with some studies showing stronger associations in males than in females. Therefore, we conducted analyses stratified by sex to investigate whether the associations differed between males and females.

To determine whether new social isolation was associated with depressive symptoms during the COVID-19 pandemic, multivariate-adjusted odds ratios (AOR) and 95% confidence intervals (CI) were calculated. The models were adjusted for sociodemographic, lifestyle, and health-related variables, as well as baseline (pre-pandemic) depressive symptoms. Interaction terms were created between sex and social isolation, and between house damage and social isolation. Logistic regression models including both main effects and interaction terms (e.g., house damage, social isolation, house damage*social isolation) were used, and p-values for interaction were calculated using the Wald test.

To examine the potential moderating role of disaster exposure, participants were stratified according to house damage due to the GEJE, which was selected as it represents a direct and substantial impact of the earthquake on participants’ lives. Stratified analyses allow for a clear presentation of associations within groups with and without housing damage, rather than focusing solely on statistical interaction.

As a sensitivity analysis to check the robustness of the main findings, participants were also stratified according to baseline depressive symptoms to examine whether pre-pandemic depressive symptoms influenced the association between new social isolation and depressive symptoms during the COVID-19 pandemic.

All statistical analyses were conducted using SPSS version 25.0 for Windows (IBM, Tokyo, Japan), and all statistical tests were two-sided. Statistical significance was set at *P* < 0.05.

## Results

### Participant characteristics by social isolation status before the COVID-19 pandemic

Men with new social isolation were younger, had no exercise habits, lacked social capital, had depressive symptoms, and had insomnia, compared with those without. Women with new social isolation were younger, had no exercise habits, were working, lacked social capital, had depressive symptoms, had insomnia, and had no house damage, compared with those without (Table 1).

**Table 1:** Participant characteristics before the COVID-19 pandemic

		Men (*n* = 2,881)			Women (*n* = 5,766)		
		Persistent non-social isolation	Social isolation from non-social isolation	*P* value	Persistent non-social isolation	Social isolation from non-social isolation	*P* value
Number		2,316	565		4,847	919	
Age (continuous)		66.9 (10.0)	65.8 (10.3)	0.029	63.7 (10.5)	60.8 (11.4)	< 0.001
BMI (%)	< 18.5 kg/m^2^	1.5	2.1	0.176	6.2	7.2	0.506
	18.5 to < 25 kg/m^2^	59.7	62.7		66.8	65.8	
	≥ 25 kg/m^2^	38.9	35.2		27.0	27.0	
Education level (%)	Elementary and junior high school	22.6	22.8	0.432	18.1	16.4	0.386
	High school	50.2	47.3		46.6	49.4	
	University degree	26.3	28.5		34.6	33.6	
	Others	0.9	1.4		0.7	0.5	
Marital status (%)	Unmarried	13.8	15.3	0.358	25.1	23.0	0.173
Number of household members (%)	Living alone	6.5	7.5	0.397	11.8	10.4	0.226
Smoking habits (%)	Smoker	20.1	20.2	0.984	3.4	3.6	0.726
Drinking habits (%)	Drinker	74.4	73.6	0.724	32.4	33.4	0.533
Exercise habits (%)	No	11.7	15.0	0.031	11.6	14.5	0.016
Working status (%)	Not working	41.7	43.4	0.473	52.3	47.0	0.003
Social capital (%)	Low	0.4	1.9	< 0.001	0.5	1.7	< 0.001
Depressive symptoms (%)		13.7	21.8	< 0.001	20.4	32.4	< 0.001
Insomnia (%)		14.9	21.4	< 0.001	23.4	29.7	< 0.001
House damage (%)	Damaged	38.2	34.3	0.091	36.7	31.2	0.002

Social isolation, LSNS-6 < 12; depressive symptoms, CES-D ≥ 16; insomnia, AIS ≥ 6

*LSNS-6* Lubben Social Network Scale-6, *BMI* body mass index, *CES-D* Center for Epidemiologic Studies Depression Scale, *AIS* Athene Insomnia Scale, *COVID-19* 2019 coronavirus disease, *GEJE* Great East Japan Earthquake

Statistical significance, *P* < 0.05

### Participant characteristics by social isolation status during the COVID-19 pandemic

While men and women with new social isolation had many similar characteristics, more women than men were married, lived with two or more people at home, had the same opportunities to see others and spend time with family, had more arguments with family members, and shopped less frequently during the pandemic compared with before the pandemic (Table 2). The distributions of depressive symptoms and social isolation at each time point (before and during the COVID-19 pandemic) are shown in Supplementary Tables 2 and 3 (Additional File 1), respectively.

**Table 2:** Participant characteristics during the COVID-19 pandemic

		Men (*n* = 2,881)			Women (*n* = 5,766)		
		Persistent non-social isolation	Social isolation from non-social isolation	*P* value	Persistent non-social isolation	Social isolation from non-social isolation	*P* value
Number		2,316	565		4,847	919	
Age (continuous)		69.2 (10.2)	68.2 (10.3)	0.038	66.1 (10.6)	63.1 (11.5)	< 0.001
Marital status (%)	Unmarried	14.6	15.2	0.706	27.5	23.7	0.017
Number of household members (%)	Living alone	7.5	8.6	0.378	13.6	11.0	0.037
Smoking habits (%)	Smoker	17.9	17.3	0.729	3.2	3.9	0.281
Drinking habits (%)	Drinker	71.7	69.3	0.275	31.8	30.3	0.387
Exercise habits (%)	No	9.7	15.6	< 0.001	11.3	15.0	0.002
Social capital (%)	Low	0.5	3.4	< 0.001	0.6	1.1	0.116
Depressive symptoms (%)		13.6	21.9	< 0.001	21.7	38.3	< 0.001
Insomnia (%)		14.3	20.2	0.001	21.1	29.7	< 0.001
Refraining from going out until April 16, 2020 (%)	Not refrained	9.7	8.8	0.353	5.3	4.8	0.168
	Neither	8.9	9.9		4.7	6.1	
	Refrained	81.4	81.3		90.0	89.1	
Refraining from going out since April 17, 2020 (%)	Not refrained	5.2	8.1	0.013	2.2	1.5	0.005
	Neither	5.8	6.9		3.3	5.3	
	Refrained	89.0	85.0		94.4	93.1	
Refraining from going out since July 29, 2020 (%)	Not refrained	4.4	6.7	0.044	1.6	1.6	< 0.001
	Neither	4.9	5.7		3.0	6.0	
	Refrained	90.7	87.6		95.4	92.4	
Opportunities to meet others face-to-face (%)	Decreased	63.3	63.2	0.928	77.1	73.6	0.012
	Neither	32.3	32.7		19.2	23.4	
	Increased	4.4	4.1		3.7	3.0	
Spending time with family (%)	Decreased	5.0	5.9	0.385	8.8	9.1	0.009
	Neither	50.6	52.8		46.1	51.2	
	Increased	44.3	41.3		45.1	39.7	
Number of times arguing with family members (%)	Decreased	10.6	9.7	0.366	13.2	10.4	0.014
	Neither	79.0	77.9		76.5	77.1	
	Increased	10.3	12.3		10.2	12.5	
Opportunities for SNS and phone calls (%)	Decreased	6.1	7.7	0.013	5.0	6.2	< 0.001
	Neither	73.1	76.7		64.5	70.4	
	Increased	20.9	15.6		30.5	23.4	
Frequency of shopping (%)	Decreased	43.1	42.5	0.585	33.1	38.5	0.002
	Neither	50.2	49.6		62.7	56.5	
	Increased	6.7	8.0		4.2	5.0	
Change in income (%)	Decreased	30.8	30.1	0.552	24.8	28.0	0.133
	Neither	66.0	65.7		71.9	68.9	
	Increased	3.2	4.2		3.3	3.1	

Social isolation, LSNS-6 < 12; depressive symptoms, CES-D ≥ 16; insomnia, AIS ≥ 6

*LSNS-6* Lubben Social Network Scale-6, *CES-D* Center for Epidemiologic Studies Depression Scale, *AIS* Athene Insomnia Scale, *COVID-19* 2019 coronavirus disease, *SNS* social networking services

Statistical significance, *P* < 0.05

Although our study focused on social isolation during the COVID-19 pandemic, previous research has shown that past disaster experience, such as the GEJE, was associated with an increased risk of depressive symptoms [20, 21]. This suggests that disaster experience may have long-term effects on mental health, potentially interacting with social isolation during subsequent crises.

### Depressive symptoms and social isolation during the COVID-19 pandemic

Both men and women with new social isolation during the COVID-19 pandemic had significantly higher prevalence rates of depressive symptoms than those without (men: AOR 1.53 [CI: 1.16–2.03]; women: AOR 1.97 [CI: 1.65–2.35]). There was no significant interaction between sex and social isolation on the odds of experiencing depressive symptoms (*P* = 0.080) (Table 3).

**Table 3:** Adjusted ORs (95% CI) for the association between depressive symptoms and new social isolation during the COVID-19 pandemic

			Crude Model		Model 1		Model 2		
		Cases/Participants	OR (95%CI)	*P* value	OR (95%CI)	*P* value	OR (95%CI)	*P* value	*P* for interaction
Men	Persistent non-social isolation	315/2,316	1.00		1.00		1.00		0.080
	Social isolation from non-social isolation	124/565	1.79 (1.42–2.25)	< 0.001	1.53 (1.15–2.02)	0.003	1.53 (1.16–2.03)	0.003	
Women	Persistent non-social isolation	1,051/4,847	1.00		1.00		1.00		
	Social isolation from non-social isolation	352/919	2.24 (1.93–2.60)	< 0.001	1.96 (1.64–2.34)	< 0.001	1.97 (1.65–2.35)	< 0.001	

*OR* odds ratio, *95% CI* 95% confidence interval

Depressive symptoms, CES-D ≥ 16; social isolation, LSNS-6 < 12

Model 1: Adjusted for age, BMI, education level, marital status, number of household members, smoking habits, drinking habits, exercise habits, working status, social capital, insomnia, refraining from going out since July 29, 2020,and depressive symptoms before the COVID-19 pandemic

Model 2: Model 1 + house damage

Statistical significance, *P* < 0.05

### Depressive symptoms and new social isolation during the COVID-19 pandemic by presence or absence of GEJE-related house damage

Men with new social isolation during the COVID-19 pandemic had a significantly higher prevalence of depressive symptoms than those without, regardless of the presence or absence of GEJE-related house damage (undamaged: AOR 1.46 [CI: 1.02–2.07]; damaged: AOR 1.75 [CI: 1.10–2.80]). The results were similar to those among women (undamaged: AOR 2.06 [CI: 1.66–2.55]; damaged: AOR 1.79 [CI: 1.30–2.47]). There was no significant interaction between housing damage due to the GEJE and social isolation on the odds of experiencing depressive symptoms (men, *P* = 0.392; women, *P* = 0.442) (Table 4). Analysis of the association between depressive symptoms and new social isolation during the COVID-19 pandemic according to age group is shown in Supplementary Table 4 (Additional File 1).

**Table 4:** Adjusted ORs (95% CI) for the association between depressive symptoms and new social isolation by presence or absence of house damage due to GEJE during the COVID-19 pandemic

				Crude Model		Adjusted Model		
			Cases/Participants	OR (95%CI)	*P* value	OR (95%CI)	*P* value	*P* for interaction
Men	Undamaged	Persistent non-social isolation	189/1,432	1.00		1.00		0.392
		Social isolation from non-social isolation	74/371	1.64 (1.22–2.21)	0.001	1.46 (1.02–2.07)	0.038	
	Damaged	Persistent non-social isolation	126/884	1.00		1.00		
		Social isolation from non-social isolation	50/194	2.09 (1.44–3.03)	< 0.001	1.75 (1.10–2.80)	0.019	
Women	Undamaged	Persistent non-social isolation	631/3,070	1.00		1.00		0.442
		Social isolation from non-social isolation	233/632	2.26 (1.88–2.71)	< 0.001	2.06 (1.66–2.55)	< 0.001	
	Damaged	Persistent non-social isolation	420/1,777	1.00		1.00		
		Social isolation from non-social isolation	119/287	2.29 (1.77–2.97)	< 0.001	1.79 (1.30–2.47)	< 0.001	

*OR* odds ratio, *95% CI* 95% confidence interval

Depressive symptoms, CES-D ≥ 16; social isolation, LSNS-6 < 12

Adjusted for age, BMI, educational level, marital status, number of household members, smoking habits, drinking habits, exercise habits, working status, social capital, insomnia, refraining from going out since July 29, 2020, and depressive symptoms before the COVID-19 pandemic

Interaction: house damage*social isolation

Statistical significance, *P* < 0.05

### Depressive symptoms and new social isolation during the COVID-19 pandemic by presence or absence of pre-pandemic depressive symptoms

Among men, new social isolation during the pandemic was associated with an increased risk of developing depressive symptoms among those without pre-pandemic depressive symptoms (AOR 1.91 [CI: 1.33–2.74]), but not among those with pre-pandemic depressive symptoms (AOR 1.01, 95% CI: 0.62–1.66). In contrast, among women, new social isolation during the COVID-19 pandemic was associated with depressive symptoms regardless of pre-pandemic depressive symptoms (low prevalence of depressive symptoms before the COVID-19 pandemic: AOR 1.76 [CI: 1.39–2.23], high prevalence of depressive symptoms before the COVID-19 pandemic: AOR 2.01 [CI: 1.47–2.76]). An interaction by pre-pandemic depressive symptoms was significant among men (*P* = 0.038), but not among women (*P* = 0.518) (See Supplementary Table 5, Additional File 1).

## Discussion

This study showed that, at the time of assessment, new social isolation during the COVID-19 pandemic was associated with depressive symptoms, compared with persistent non-social isolation. Our results were consistent with those of previous studies of social isolation during the COVID-19 pandemic [17, 18]. Additionally, while our findings were obtained in Japan, a developed Asian country, the COVID-19 pandemic also had severe impacts on developing Asian countries. For example, a study from Vietnam reported substantial occupational and economic hardships during the first national lockdown [30]. This contrast suggests that the nature of the challenges may differ, with psychosocial issues such as social isolation and depressive symptoms being more prominent in developed countries, and economic vulnerability being more evident in developing countries. Addressing these different vulnerabilities requires context-specific public health strategies. Particularly, our findings suggest that public health support to prevent social isolation during a pandemic may help address depressive symptoms.

We hypothesized that past disaster experience possibly affected the association between new social isolation and depressive symptoms during the COVID-19 pandemic. However, this association was not affected by past disaster experience. The direct impact of the GEJE against the COVID-19 pandemic may have reduced, as it has been almost 10 years since the GEJE incidence. Feelings of grief and loss have been shown to subside over time, leading to acceptance of reality [31]. Even if the reality was harsh at the time of the GEJE, the passage of more than 10 years may have triggered a mind shift towards accepting the reality of the disaster, and the effect may have reduced. Therefore, the level of vulnerability due to the disaster may have decreased, and its impact on social isolation and depressive symptoms stemming from a pandemic such as this may have been insignificant.

In the present study, among men, new social isolation during the COVID-19 pandemic was associated with a high prevalence of depressive symptoms among those without pre-pandemic depressive symptoms. In addition, the rate of social isolation among men increased during compared with before the COVID-19 pandemic [12]. A study of 434 adult Canadian men showed that the rate of depressive symptoms increased with fewer social connections [32]. Similar results were confirmed in a study of a Chinese population [33]. A sense of disconnection from others due to the loss of human connection has been noted to increase vulnerability to depressive symptoms [34, 35]. For men, work and leisure activities constitute their primary social connections [32]. Although they may appear connected to others, they are more likely to feel alienated in their relationships and workplaces, leading to social isolation [36]. To prevent COVID-19 spread, people avoided unnecessary trips out of the house, and teleworking was introduced to the workplace, resulting in a sharp decrease in opportunities for human contact. Even if they had no experience of depressive symptoms before the COVID-19 pandemic, the behavioral restrictions imposed by the pandemic may have made them more likely to feel alienated, leading to social isolation and the development of depressive symptoms.

Among women, new social isolation was associated with depressive symptoms, regardless of their status before the COVID-19 pandemic. This finding was consistent with previous findings that social isolation owing to COVID-19-related restrictions was associated with depressive symptoms [37–39]. Women have been found to experience higher levels of mental illness than men in general [40] and during the COVID-19 pandemic [41–47]. Decreased level of social contact has been associated with mental health decline and increased prevalence of depressive symptoms [48, 49]. In the present study, women with prior experience of depressive symptoms may have been at a higher risk of developing new depressive symptoms during the COVID-19 pandemic because of mental health decline, as they became less connected to others and experienced social isolation. In addition, women with no prior experience of depressive symptoms may have experienced social isolation and worsened mental health as a result of reduced social contact, which may have increased their risk of developing depressive symptoms. 

This study has some limitations. First, the participants may have had greater health awareness and better health status than the target population, as they voluntarily participated in the health surveys. Additionally, this study only included participants who were involved during both the pre- and pandemic periods, and those lost to follow-up or with missing values may have had higher chances of experiencing social isolation or depressive symptoms, potentially leading to systematic bias. Therefore, the prevalence of depressive symptoms may have been underestimated, and generalization of the results must be carefully considered. Second, there is a possibility of reverse causality, such as depressive symptoms leading to social isolation or isolation resulting from the inability to leave home owing to being ill. Nevertheless, even after excluding individuals with depressive symptoms at baseline, the association between new social isolation and the development of depressive symptoms persisted in the additional analyses, supporting the robustness of our findings. Third, as our data were collected using self-administered questionnaires, measurement errors, including recall bias, may be present. The potential impact of common method bias should be acknowledged, since both social isolation and depressive symptoms were assessed using the same survey method. This study is important because it involved the use of a population-based cohort design and a large sample size to examine whether the association between new social isolation and depressive symptoms during the COVID-19 pandemic was influenced by past disaster experience and/or presence of depressive symptoms.

## Conclusions

Our study showed that new social isolation during the COVID-19 pandemic was associated with depressive symptoms, regardless of past disaster experience. Our findings also showed that, among those without prior experience of depressive symptoms, new social isolation during the COVID-19 pandemic was associated with the development of depressive symptoms. To reduce the prevalence of depressive symptoms during a major infectious disease pandemic such as the COVID-19 pandemic, it is important to adopt a public health perspective to prevent social isolation, regardless of the presence or absence of past disaster experience.

## Supplementary Information

Additional file 1: Supplementary Table S1. Correlation coefficients of behaviors related to the COVID-19 pandemic. Supplementary Table S2: Distribution of change in depressive symptoms. Supplementary Table S3. Distribution of change in social isolation. Supplementary Table S4. Adjusted ORs for the association between depressive symptoms and new social isolation by age group during the COVID-19 pandemic. Supplementary Table S5. Adjusted ORs for the association between depressive symptoms and new social isolation during the COVID-19 pandemic according to the presence of depressive symptoms pre-COVID-19

## Data Availability

TMM cohort data are available for a fee to researchers in Japan who have been approved by the prescribed registration and review procedures. For more information, visit http://www. dist. megabank. tohoku. ac. jp/.

## Abbreviations

AOR: adjusted odds ratio
CES-D: Center for Epidemiological Studies-Depression Scale
CI: confidence interval
COVID-19: coronavirus disease 2019
GEJE: Great East Japan Earthquake
LSNS-6: Lubben Social Network Scale
WHO: World Health Organization
