# Supplementary material for: Influence of housing damage caused by the Great East Japan Earthquake on the association between new social isolation and depressive symptoms during the COVID-19 pandemic: findings from the Tohoku Medical Megabank Community-Based Cohort Study
Source: BMC Public Health. 2025 Dec 24;26:342. doi: 10.1186/s12889-025-25996-9 (PMC12837444; doi:10.1186/s12889-025-25996-9)
Supplement: Supplementary file 1 — Additional file 1: Supplementary Table S1. Correlation coefficients of behaviors related to the COVID-19 pandemic. Supplementary Table S2: Distribution of change in depressive symptoms. Supplementary Table S3. Distribution of change in social isolation. Supplementary Table S4. Adjusted ORs for the association between depressive symptoms and new social isolation by age group during the COVID-19 pandemic. Supplementary Table S5. Adjusted ORs for the association between depressive symptoms and new social isolation during the COVID-19 pandemic according to the presence of depressive symptoms pre-COVID-19 [file 12889_2025_25996_MOESM1_ESM.docx]

**Supplementary Table 1.** Correlation coefficients of behaviors related to the COVID-19 pandemic

|  | Refraining from going out until April 16, 2020 | Refraining from going out since April 17, 2020 | Refraining from going out since July 29, 2020 | Opportunities to meet others face-to-face | Spending time with family | Number of times arguing with family members | Opportunities for SNS and phone calls | Frequency of shopping | Change in income |
| --- | --- | --- | --- | --- | --- | --- | --- | --- | --- |
| Refraining from going out until April 16, 2020 |  | 0.56** | 0.44** | 0.15** | 0.15** | 0.04* | 0.07** | 0.15** | 0.03 |
| Refraining from going out since April 17, 2020 |  |  | 0.63** | 0.18** | 0.17** | 0.03 | 0.06** | 0.17** | 0.02 |
| Refraining from going out since July 29, 2020 |  |  |  | 0.16** | 0.15** | 0.04* | 0.03 | 0.15** | 0.03 |
| Opportunities to meet others face-to-face |  |  |  |  | 0.28** | 0.15** | 0.15** | 0.20** | 0.06** |
| Spending time with family |  |  |  |  |  | 0.23** | 0.15** | 0.23** | 0.09** |
| Number of times arguing with family members |  |  |  |  |  |  | 0.14** | 0.17** | 0.08** |
| Opportunities for SNS and phone calls |  |  |  |  |  |  |  | 0.15** | 0.08** |
| Frequency of shopping |  |  |  |  |  |  |  |  | 0.11** |
| Change in income |  |  |  |  |  |  |  |  |  |

****** Statistically significant at *P* < 0.01.

* Statistically significant at *P* < 0.05.

COVID-19, coronavirus disease 2019; SNS, social networking service

**Supplementary Table 2.** Distribution of depressive symptoms before and during the COVID-19 pandemic

|  |  | During COVID-19 pandemic | | *P* value |
| --- | --- | --- | --- | --- |
|  |  | Depressive  Symptoms – | Depressive  Symptoms + |  |
| Before COVID-19 | Depressive  symptoms – | Persistent low prevalence of depressive symptoms  n = 8,085 (65.2%) | High prevalence of depressive symptoms from low prevalence of depressive symptoms  n = 1,183 (9.5%) | <0.001 |
|  | Depressive  symptoms + | Low prevalence of depressive symptoms from high prevalence of depressive symptoms  n = 1,094 (8.8%) | Persistent high prevalence of depressive symptoms  n = 2,030 (16.4%) |  |

Statistical significance was set at *P* < 0.05. COVID-19, coronavirus disease 2019

**Supplementary Table 3.** Distribution of social isolation before and during the COVID-19 pandemic

|  |  | During COVID-19 pandemic | | *P* value |
| --- | --- | --- | --- | --- |
|  |  | Non-social isolation | Social isolation |  |
| Before COVID-19 | Non-social isolation | Persistent non-social isolation  n = 7,163 (57.8%) | Social isolation from  non-social isolation  n = 1,484 (12.0%) | <0.001 |
|  | Social isolation | Non-social isolation from social isolation  n = 951 (7.7%) | Persistent social isolation  n = 2,794 (22.5%) |  |

Statistical significance was set at *P* < 0.05. COVID-19, coronavirus disease 2019

**Supplementary Table 4.** Adjusted ORs for the association between depressive symptoms and new social isolation by age group during the COVID-19 pandemic

|  |  |  |  | Crude Model | |  | Adjusted Model* | |  |
| --- | --- | --- | --- | --- | --- | --- | --- | --- | --- |
|  |  |  | Cases / Participants | OR (95%CI) | *P* value |  | OR (95%CI) | *P* value | *P* for interaction |
| Men | <65 years | Persistent non-social isolation | 78 / 624 | 1.00 |  |  | 1.00 |  | 0.540 |
|  |  | Social isolation from non-social isolation | 44 / 178 | 2.30  (1.52-3.48) | <0.001 |  | 1.77  (1.04-3.02) | 0.035 |  |
|  | ≥65 years | Persistent non-social isolation | 237 / 1692 | 1.00 |  |  | 1.00 |  |  |
|  |  | Social isolation from non-social isolation | 80 / 387 | 1.60  (1.21-2.12) | 0.001 |  | 1.42  (1.02-1.99) | 0.041 |  |
| Women | <65 years | Persistent non-social isolated | 467 / 2,083 | 1.00 |  |  | 1.00 |  | 0.821 |
|  |  | Social isolation from non-social isolation | 203 / 501 | 2.36  (1.92-2.90) | <0.001 |  | 2.08  (1.63-2.65) | <0.001 |  |
|  | ≥65 years | Persistent non- social isolation | 584 / 2,764 | 1.00 |  |  | 1.00 |  |  |
|  |  | Socially isolated from non-social isolation | 149 / 418 | 2.07  (1.66-2.58) | <0.001 |  | 1.87  (1.44-2.43) | <0.001 |  |

OR, odds ratio; 95% CI, 95% confidence interval; COVID-19, coronavirus disease 2019

Depressive symptoms, CES-D ≥ 16; social isolation, LSNS-6 < 12.

* Adjusted for BMI, educational level, marital status, number of household members, smoking habits, drinking habits, exercise habits, working status, social capital, insomnia, refraining from going out since July 29, 2020, and depressive symptoms before the COVID-19 pandemic.

Interaction: house damage*social isolation.

Statistical significance was set at *P* < 0.05.

**Supplementary Table 5.** Adjusted ORs for the association between depressive symptoms and new social isolation during the COVID-19 pandemic according to the presence of depressive symptoms pre-COVID-19

|  |  |  |  | Crude Model | | Model 1 | | Model 2 | | Model 3 | |  |
| --- | --- | --- | --- | --- | --- | --- | --- | --- | --- | --- | --- | --- |
|  |  |  | Cases / Participants | OR (95%CI) | *P* value | OR (95%CI) | *P* value | OR (95%CI) | *P* value | OR (95%CI) | *P* value | *P* for interaction |
| Men | Without depressive symptoms | Persistent non-social isolation | 143 / 1,998 | 1.00 |  | 1.00 |  | 1.00 |  | 1.00 |  | 0.038 |
|  |  | Social isolation from non-social isolation | 53 / 442 | 1.77  (1.27-2.47) | 0.001 | 1.82  (1.29-2.58) | 0.001 | 1.83  (1.29-2.58) | 0.001 | 1.91  (1.33-2.74) | <0.001 |  |
|  | With depressive symptoms | Persistent non-social isolation | 172 / 318 | 1.00 |  | 1.00 |  | 1.00 |  | 1.00 |  |  |
|  |  | Social isolation from non-social isolation | 71 / 123 | 1.16  (0.76-1.76) | 0.491 | 1.10  (0.69-1.73) | 0.688 | 1.10  (0.69-1.74) | 0.688 | 1.01  (0.62-1.66) | 0.966 |  |
| Women | Without depressive symptoms | Persistent non-social isolation | 472 / 3,860 | 1.00 |  | 1.00 |  | 1.00 |  | 1.00 |  | 0.518 |
|  |  | Social isolation from non-social  isolation | 127 / 621 | 1.85  (1.49-2.29) | <0.001 | 1.84  (1.47-2.30) | <0.001 | 1.84  (1.47-2.31) | <0.001 | 1.76  (1.39-2.23) | <0.001 |  |
|  | With depressive symptoms | Persistent non-social isolation | 579 / 987 | 1.00 |  | 1.00 |  | 1.00 |  | 1.00 |  |  |
|  |  | Social isolation from non-social  isolation | 225 / 298 | 2.17  (1.62-2.91) | <0.001 | 2.16  (1.59-2.93) | <0.001 | 2.18  (1.61-2.96) | <0.001 | 2.01  (1.47-2.76) | <0.001 |  |

OR, odds ratio; 95% CI, 95% confidence interval; COVID-19, coronavirus disease 2019.

Depressive symptoms, CES-D ≥ 16; social isolation, LSNS-6 < 12.

Model 1 was adjusted for age, BMI, education level, marital status, number of household members, smoking habits, drinking habits, exercise habits, working status, social capital, and insomnia.

Model 2: Model 1+ house damage.

Model 3: Model 2 + refraining from going out since July 29, 2020, + opportunities to meet others face-to-face + spending time with family + number of times arguing with family members + opportunities for SNS and phone calls + frequency of shopping + change in income.

Interaction: Depressive symptoms before COVID-19 pandemic × social isolation.

Statistical significance was set at *P* < 0.05.
